# The Role of Exercise in Regulating the Generation of Extracellular Vesicles in Cardiovascular Diseases

**DOI:** 10.31083/j.rcm2511392

**Published:** 2024-11-04

**Authors:** Peng Shen, Yue Qiu, Yan-Yan Sun, Yue-Ying Jiang, Xiu-Mei Guan, Min Cheng, Yan-Xia Wang

**Affiliations:** ^1^School of Rehabilitation Medicine, Shandong Second Medical University, 261053 Weifang, Shandong, China; ^2^School of Basic Medical Sciences, Shandong Second Medical University, 261053 Weifang, Shandong, China

**Keywords:** EVs, exercise, cardiovascular diseases, miRNA

## Abstract

Extracellular vesicles (EVs) are nanoscale vesicles released by cells, which play an important role in intercellular communication by transporting proteins, lipids, nucleic acids, and other molecules. Different intensities of exercise can induce the release of EVs from cells and tissues, such as endothelial cells, skeletal muscle and adipose tissue, hepatocytes, immune cells, and neuronal cells. Exercise-induced EVs exert cardiovascular protective effects such as anti-inflammatory and anti-oxidative by altering their contents. This paper reviews the cell and tissue sources of EVs induced by exercise of different intensities, the regulatory effects of different exercise intensities on EVs, and their mechanisms of action in cardiovascular diseases. The aim is to provide new insights for the treatment of cardiovascular diseases and offer scientific evidence for the construction of engineered EVs mimicking the effects of exercise.

## 1. Introduction

The prevalence of cardiovascular diseases is continually increasing, which has become the primary cause of disease-related mortality [1]. Therefore, the prevention and treatment of cardiovascular diseases are of urgent importance. As a primary strategy for non-invasive proactive health maintenance and cardiovascular disease prevention, exercise stimulates the release of extracellular vesicles (EVs) from various tissues and cells, including endothelial cells, skeletal muscle, and adipose tissue [2, 3]. EVs are small vesicles with a lipid bilayer membrane structure, containing contents such as proteins, lipids, nucleic acids, and metabolites, which allow for intercellular communication [4, 5]. At present, EVs are considered to be important mediators of communication in regulating cardiovascular health through exercise [6]. Studies have shown that exercise of appropriate intensity can regulate the contents of EVs, exerting anti-inflammatory and anti-oxidative effects, enhancing angiogenesis, alleviating myocardial injury, and improving endothelial function. These effects contribute to the effective prevention and treatment of cardiovascular diseases [7, 8, 9]. Conversely, EVs generated from inappropriate exercise intensity may induce inflammatory and oxidative stress reactions, thus compromising cardiovascular function [10, 11, 12]. This review provides a comprehensive discussion of the influence of exercise of varying intensities on the secretion of EVs as well as the regulatory effects and mechanisms of EVs secreted by different intensity exercises on cardiovascular diseases. The aim is to provide a theoretical basis for the promotion of cardiovascular health through exercise.

## 2. EVs

EVs are tiny vesicles released by cells into the microenvironment, with diameters ranging from approximately 35 nm to 1 µm. Based on their origin and biogenesis, EVs can be classified into microvesicles, exomeres, exosomes, and apoptotic bodies [5, 13]. Microvesicles, also referred to as microparticles, are anucleate vesicular clusters originating from the plasma membrane ranging from 50 nm to 1 µm. These microparticles can be shed directly from the cell surface through mechanisms involving calcium influx, membrane reorganization, and cytoskeletal remodeling. They perform functions by conveying specific information to target cells [5, 14, 15, 16]. Exomeres are non-vesicular nanoparticles with a diameter of approximately 35 nm, composed of various lipids and rich in metabolic enzymes, participating in multiple metabolic processes within the body [17, 18]. Exosomes are lipid bilayer vesicles that originate from endosomes, with a diameter ranging from approximately 40 nm to 160 nm (average about 100 nm). During the formation of exosomes, the cytoplasmic membrane undergoes an initial invagination to form early-sorting endosomes (ESEs). Following maturation, ESEs develop into late-sorting endosomes (LSEs), which ultimately evolve into multivesicular bodies (MVBs). MVBs release exosomes into the extracellular environment by fusing with the cytoplasmic membrane [19, 20, 21, 22]. Apoptotic bodies are defined as vesicular structures, which derive from apoptotic cells through two distinct mechanisms: germination shedding and autophagosome formation. Based on their diameter, apoptotic bodies can be categorized into two distinct groups: small apoptotic bodies (100 nm–1 µm) and large apoptotic bodies (1 µm–5 µm) [23]. Increasing evidence indicates that exercise, as a critical physical stimulus, can promote the release of EVs into the circulatory system and then regulate cardiovascular function.

## 3. Cell and Tissue Sources of Exercise-Modulated EVs Release

### 3.1 Endothelial Cells

Endothelial cells are situated on the innermost layer of the vascular wall. They can detect mechanical force alterations induced by exercise, such as blood flow shear stress and wall tension through membrane sensors or receptors, and then regulate the release of exosomes [24, 25]. A study of mice carried out 4 weeks of swimming and athletes underwent 1 year of rowing indicated that exercise promoted the release of exosomes enriched with miR-342-5p from endothelial cells. These exosomes enhanced the phosphorylation of protein kinase B (Akt) in cardiomyocytes by inhibiting the apoptotic pathways of caspase-9 and c-Jun N-terminal kinase 2 (Jnk2) and targeting protein phosphatase 1F (PPM1F). This process ultimately increased the anti-apoptotic capability of cardiomyocytes and then protected the heart from myocardial ischemic injury [26]. Additionally, exercise can regulate cardiovascular function by stimulating the release of EVs from endothelial progenitor cells (EPCs). EPCs are precursor cells of endothelial cells, which can differentiate into mature endothelial cells to promote the repair of damaged blood vessels. Ma *et al*. [27] found that exosomes released from EPCs after 4 weeks of treadmill exercise in mice protected vascular endothelial cells from impairment caused by high glucose and hypoxic conditions. Moreover, 4 weeks of treadmill exercise before stroke modeling in mice accelerated post-stroke recovery via promoting the release of a large number of exosomes from EPCs [28].

### 3.2 Skeletal Muscle

Skeletal muscle, the primary executor of physical activity, can release EVs containing various signaling regulators into the circulatory system. Appropriate exercise can induce changes in the content of EVs released from skeletal muscle cells, thereby modulating inflammatory and oxidative stress responses in the cardiovascular system, as well as systemic glucose and lipid metabolism. These ultimately reduce the incidence and progression of cardiovascular disease [11, 29, 30, 31, 32]. Guescini *et al*. [4] conducted a study in which they analyzed blood samples from healthy individuals who had just completed an acute exercise session. Their findings revealed that exercise stimulated skeletal muscle to secrete α-sarcoglycan-positive EVs carrying miR-181a-5p, which in turn inhibited myocardial inflammation and oxidative stress, thereby preventing the onset of cardiovascular diseases [33]. Barone *et al*. [34] observed that mice subjected to 6 weeks of endurance exercise demonstrated enhanced rapid secretion of exosomes containing heat shock protein 60 (HSP60) from skeletal muscle into the circulatory system, thus increasing mitochondrial activity in cardiomyocytes and providing a protective effect on these cells. Moreover, research demonstrated that 16 weeks of swimming training stimulated the secretion of EVs from skeletal muscle, which improved glucose tolerance in obese mice and apolipoprotein E knock out (*ApoE*^-/-^) mice, reduced visceral lipid accumulation, alleviated liver damage, as well as inhibited the development of atherosclerosis [35]. Furthermore, exercise has been demonstrated to decelerate vascular aging in mice by stimulating the release of EVs from skeletal muscle, which are enriched with fibronectin type III domain-containing protein 5 (FNDC5)/irisin. This process concurrently reduces oxidative stress, inflammation, and endothelial dysfunction induced by angiotensin II, thereby exerting a protective effect on the cardiovascular system [36, 37].

### 3.3 Adipose Tissue

Adipose tissue, which encompasses both white and brown adipose tissues, represents the body’s largest endocrine organ. It regulates cardiovascular homeostasis through the secretion of a series of cytokines [8]. Dysfunction of white adipose tissue can result in the onset of type II diabetes and cardiovascular complications. Conversely, brown adipose tissue plays a positive role in the physiological regulation of the cardiovascular system [8]. Research indicated that exercise promoted the browning of white adipose tissue and increased the volume of brown adipose tissue [38]. Concurrently, exercise can exert anti-apoptotic effects on cardiomyocytes by altering the content of EVs secreted by adipose tissue. The 4 weeks of swimming exercise promoted the secretion of EVs enriched with miR-125b-5p, miR-128-3p, and miR-30d-5p from brown adipose tissue in mice to inhibit the pro-apoptotic mitogen-activated protein kinase (MAPK) signaling pathway and then protect the heart from myocardial ischemia-reperfusion (MI/R) injury [8].

### 3.4 Hepatocytes

The liver is responsible for the synthesis, metabolism, and redistribution of nutrients within the human body, playing a crucial role in the overall metabolic process [39]. Li *et al*. [40] and Zhao *et al*. [41] have demonstrated that EVs secreted by hepatocytes containing let-7b-5p can reduce mitochondrial oxidative phosphorylation and inhibit the conversion of white adipose tissue to brown adipose tissue, thereby promoting the development of obesity. Furthermore, research has indicated that EVs synthesized and secreted by the liver play a pivotal role in the regulation of cardiovascular diseases, including atherosclerosis, coronary artery disease, thrombosis, and myocardial infarction [42, 43]. In a murine model of nonalcoholic fatty liver disease, EVs derived from steatotic hepatocytes have been observed to enhance coronary microvascular permeability by modulating the novel miR-7/lysosomal associated membrane protein 1 (LAMP1)/Cathepsin B/NOD-like receptor family pyrin domain-containing 3 (NLRP3) inflammasome pathway which in turn disrupts the integrity of the microvascular endothelial barrier, leading to the occurrence of abnormal coronary blood flow reserve [44, 45]. Additionally, Lou e*t al.* [46] found that exercise promoted the enrichment of miR-122-5p in liver-derived EVs. By targeting endothelial cells through 1-acyl-sn-glycerol-3-phosphate acyltransferase (AGPAT1), miR-122-5p enhances the utilization of fatty acids by endothelial cells, thereby increasing angiogenesis. In conclusion, the release of liver-derived EVs is subject to regulation by exercise, and these EVs play a role in the regulation of metabolism and the protection of the cardiovascular system [40, 41, 42, 43, 44, 45, 46, 47].

### 3.5 Immune Cells

Immune cells, including lymphocytes, macrophages, monocytes, and antigen-presenting cells, have the capacity to regulate the immune system and modulate cardiovascular function modulation through the release of EVs [48]. Xiong *et al*. [49] discovered that following an acute myocardial infarction, EVs released by T lymphocytes, macrophages, dendritic cells, and mast cells were involved in post-infarction immunomodulation and acted as part of myocardial repair. Moreover, following incremental cycling exercise, EVs secreted by leukocytes, lymphocytes, and antigen-presenting cells contribute to the enhancement of vascular function and immune regulation, including adaptive immunity [50]. In a study conducted by Highton *et al*. [51], renal transplant patients were observed after engaging in moderate-to-high intensity exercise. The findings indicated that the percentage of microparticles produced by intermediate monocytes decreased following exercise, which contributed to anti-thrombotic effects and protected vascular function in the transplanted kidneys. The aforementioned studies suggest that exercise can promote exercise adaptation and improve cardiovascular function by regulating the release of EVs from immune cells.

### 3.6 Neuronal Cells

Neuronal cells are a vital component of the nervous system [52]. Neurons that release EVs facilitate intercellular communication, process components of unwanted neuronal activity, and propagate pathological factors in neurodegenerative disease [53, 54]. In a study conducted by Zumkehr *et al*. [55], it was found that exosomes secreted by neuronal cells containing miR-124a can be absorbed by primary astrocytes, resulting in elevated intracellular levels of miR-124a and enhanced expression of glutamate transporter 1. This mechanism assists in maintaining synaptic glutamate homeostasis and prevents neuronal excitotoxicity. Meanwhile, Luo *et al*. [56] discovered that miR-150-3p-rich exosomes secreted by neural stem cells could promote neuronal proliferation by inhibiting the caspase 2 (CASP2) signaling pathway, thus preventing brain injury. Furthermore, EVs secreted by neurons have been demonstrated to modulate the cardiovascular system. Wang *et al*. [57] demonstrated that under conditions of oxygen-glucose deprivation, neurons are capable of secreting exosomes containing lncRNA H19. These exosomes have been demonstrated to increase endothelial cell permeability through the miR-18a/vascular endothelial growth factor (VEGF) pathway, thereby disrupting the integrity of the blood-brain barrier. Katsur *et al*. [58] found that exosomes secreted by neural stem cells can delay the opening of mitochondrial permeability transition pores (mPTP) mediated by reactive oxygen species (ROS) in cardiomyocytes through the gp130/Janus tyrosine Kinase (JAK)/signal transducer and activator of transcription (STAT) pathway. This mechanism serves to safeguard cardiomyocytes from oxidative stress and to diminish the infarct size in myocardial infarction. In addition, Delgado-Peraza *et al*. [59] showed that exercise stimulates neurons to secrete EVs rich in brain-derived neurotrophic factor (BDNF), proBDNF, and humanin. This secretion has been demonstrated to enhance cognitive function in patients diagnosed with Alzheimer’s disease (AD). Indeed, EVs secreted by neural cells play an important role in neuroprotection and the regulation of the cardiovascular system.

In addition to the aforementioned tissues and cells, mesenchymal stem cell, cardiomyocytes, platelets, neurons, and antigen-presenting cells can also secrete exercise-induced EVs [8, 46, 47, 50, 59, 60, 61]. Although efforts are being made, the exact tissues and cells sources of exercise-induced EVs are not entirely clear. Endothelial cells and skeletal muscle may be the predominant sources of circulating EVs [26].

## 4. Regulation of EVs Secretion by Exercise of Different Intensities

### 4.1 Classification of Exercise Intensity Levels

According to the guidelines of the American college of sports medicine (ACSM), the intensity levels are typically classified into low, moderate, and high based on absolute and relative exercise intensity indicators in human physical activity (Table 1) [62, 63]. Absolute indicator primarily refers to metabolic equivalents (METs), which accounts for the influence of age on the classification of exercise intensity levels. Relative indicators contain percentages of maximal oxygen uptake (VO_2max_), maximal heart rate (HR_max_), heart rate reserve (HRR), and percentage of one repetition maximum (1 RM) [62, 63]. The classification of exercise intensity levels based on relative indicators does not consider the factors of age and sex. In fact, the potential influence of age and sex cannot be ignored. Females have a higher heart rate (HR) and oxygen consumption (VO_2_) than in males and older persons possess higher HR and VO_2_ than in younger individuals when they carry out the same intensity exercise [64]. Therefore, the current classification of exercise intensity levels may result in excessive exercise intensity for females and older people, who can choose the low value in the range of exercise intensity indicators. In animal experiments, the exercise intensity is usually set with reference to the Bedford exercise protocol [65], with specific grading of exercise intensity levels as referenced in Table 1.

**Table 1.** **Grading of different intensities exercise**.

	Human								Animal	
	Absolute exercise intensity (METs) in adults (age in years)				Relative exercise intensity				Mouse	Rat
Classification	20–39 yr	40–64 yr	65–79 yr	80+ yr	%VO_2max_	%HR_max_	%HRR	%1 RM	Running speed (m/min)	
Low intensity	2.4–4.7	2.0–3.9	1.6–3.1	1.1–1.9	<40	35–54	20–39	30–50	<10	<16
Moderate intensity	4.8–7.1	4.0–5.9	3.2–4.7	2.0–2.9	40–60	55–69	40–59	51–70	10–20	16–24
High intensity	7.2–10.1	6.0–8.4	4.8–6.7	3.0–4.25	>60	70–89	60–84	71–85	>20	>24

METs, metabolic equivalents; yr, year; VO_2max_, maximal oxygen uptake; HR_max_, maximal heart rate; HRR, heart rate reserve; 1 RM, one repetition maximum.

### 4.2 Modulation of EVs Secretion by Low-Intensity Exercise

A series of studies have demonstrated that both acute and chronic low-intensity exercise effectively increase the concentration of EVs in the serum of various populations or mice, but do not significantly affect the diameter of EVs [27, 66]. Just *et al*. [32] observed that following the performance of five sets of knee joint extension exercises at an intensity of 30% 1 RM with blood flow restriction in 9 healthy men, an increase in EV concentration was evident as early as 5 min post-exercise. Xhuti *et al*. [67] observed a significant increase in the plasma levels of tumor susceptibility gene (*TSG101*) in elderly individuals and young adults after 12 weeks of thrice-weekly home-based resistance training. TSG101 serves as a marker for extracellular vesicle biogenesis, indicating that 12 weeks of low-intensity resistance training augmented the concentration of EVs. Furthermore, following a single session of acute treadmill exercise intervention consisting of 40 min at a speed of 14–16 m/min in 5 Wistar rats, it was observed that low-intensity exercise did not alter the diameter of EVs in the rat serum. However, the median concentration of EVs in serum was increased from 1.1 × 10^9^/mL in sedentary rats to 3 × 10^9^/mL post-exercise [66]. Ma *et al*. [27] found that after 4 weeks of low-intensity treadmill exercise, consisting of 5 sessions per week lasting 60 min each, the number of exosomes isolated from the plasma of C57BL/6J mice was higher in the exercise group compared to the sedentary group. Nevertheless, the diameters of the exosomes isolated from the plasma of exercise and sedentary mice were 110 ± 8 nm and 112 ± 5 nm, respectively, showing no significant difference between the two groups. An 8-week regimen of low-intensity treadmill exercise in type II diabetes model db/db mice revealed that exercise promoted the release of miR-445-rich exosomes. This process downregulated matrix metallopeptidase 9 (MMP9), thus preventing myocardial fibrosis and exerting a cardioprotective effect [60].

### 4.3 Modulation of EVs Secretion by Moderate-Intensity Exercise

As with low-intensity exercise, both acute and chronic moderate-intensity exercise can also induce the generation of EVs in serum from various populations or rodents. However, they do not exert a significant impact on the diameter of exosomes. Warnier *et al*. [68] observed that the number of exosome-like vesicles (ELVs) in the plasma significantly increased in healthy subjects following 60 min of cycling exercise at an intensity of 55% VO_2max_. In another study, healthy female subjects aged 18–40 showed no change in the size of EVs in their plasma but did exhibit a significant increase in EVs quantity after 30 min of running at 59% HRR [69]. In animal experiments, following a single acute running exercise session of 40 min at a speed of 20–22 m/min, the average diameter of EVs in the serum of rats was 91.5 nm, which was not significantly different compared to the sedentary and low-intensity exercise groups. However, the concentration of EVs in the serum were significantly higher than that in the sedentary group [66]. Barcellos *et al*. [70] found that after a 12-week treadmill exercise program at 60% VO_2max_ intensity in elderly rats, there was a significant increase in the content of the EV surface marker CD63 in plasma, indicating that exercise promotes the secretion of EVs. Additionally, 4 weeks of moderate-intensity treadmill exercise prior to stroke modeling in mice promoted the secretion of EVs derived from EPCs [28].

### 4.4 Modulation of EVs Secretion by High Intensity Exercise

Currently, it is widely believed that high intensity exercise promotes the secretion of EVs in the serum/plasma of healthy individuals. After a single bout of 20 min of cycling exercise at 70% VO_2max_ intensity in healthy subjects, it was observed that the size of EVs remained unchanged, with measurements of 88 ± 4.7 nm before exercise and 89 ± 4.9 nm post-exercise. However, there was a significant increase in the concentration of EVs in their blood [71]. Meanwhile, Chong *et al*. [72] reached a consistent conclusion in healthy men subjected to the same duration and intensity of cycling exercise. Following a single 60-minute acute exercise session involving 11 healthy subjects, with an exercise intervention consisting of 30 min at 55% VO_2max_, 20 min at 70% VO_2max_, and 10 min at 80% VO_2max_, a significant increase in plasma EVs was observed four hours post-exercise [73].

However, the regulation of EV secretion in cardiovascular disease patients or individuals at risk for cardiovascular disease following high intensity exercise yields different results. Apostolopoulou *et al*. [74] conducted a 12-week high intensity interval training program (three times per week, consisting of five rounds of 4 min at 90% HR_max_ followed by 3 min at 70% HR_max_) in 20 male subjects with type 2 diabetes, 12 sedentary insulin-sensitive non-diabetic subjects, and 11 insulin-resistant non-diabetic subjects. The researchers observed that exercise significantly increased serum EV concentrations in both type 2 diabetic subjects and insulin-resistant non-diabetic subjects. However, they did not find a significant effect of exercise on the EV concentration in sedentary insulin-sensitive non-diabetic subjects, nor did they observe any effect on the size of the EVs. Dimassi *et al*. [75] demonstrated that an 8-week high intensity interval training program (three times per week, consisting of 15 min of warm-up, three bouts of 10 min at 60–80% HR_max_, with 5 min of active recovery) significantly increased endothelial microparticle levels in both normal weight and obese populations. However, a 12-week high intensity interval training regimen (three times per week, intensity at 90–95% HR_max_, with 3-minute intervals of rest, totaling 38 min of exercise and rest cycles) was found to have no effect on the levels of endothelial microparticles in stable coronary artery disease patients [76]. The differential results may be attributed to the types of disease among participants and the maximum exercise intensity in the study. It has been demonstrated that exercise at a certain intensity range can promote the generation of EVs. However, exceeding the threshold intensity may not affect EV secretion. Further investigation is required to determine the intensity threshold for exercise concerning different diseases.

In conclusion, low and moderate-intensity exercise can stimulate the secretion of EVs across different populations, while high intensity exercise can enhance EV secretion in healthy individuals. Nevertheless, for individuals with cardiovascular diseases or cardiovascular risk factors, differential outcomes may arise due to variations in disease type or the maximal exercise intensity employed. Maybe, the current classification of exercise intensity levels may result in excessive exercise intensity for individuals with cardiovascular diseases or cardiovascular risk factors in the different exercise intensity levels. At present, the majority of research is focused on the regulation of EV secretion by individual low, moderate, and high intensity exercises. The evidence regarding the regulatory patterns of EV secretion by different intensity exercises is limited and inconsistent. Oliveira *et al*. [66] found that acute low, moderate, and high intensity exercises significantly increased the concentration of EVs in rat serum, with no significant differences observed between the various intensity levels. However, Ma *et al*. [27] observed that following a 4-week treadmill exercise intervention in mice, exercise intensity exhibited a dose-dependent effect on EV secretion. Moderate-intensity exercise was found to significantly enhance EV secretion compared to low-intensity exercise. Consequently, further investigation is required to elucidate the regulation rule of EV secretion by exercise at different intensities.

Furthermore, it is noteworthy that the release of EVs by exercise exhibits a temporal effect. In healthy subjects, during 30 min of cycling exercise at 55% VO_2max_, the quantity of ELVs increased by 313% compared to pre-exercise levels, but decreased by 53% when the exercise duration was extended to 60 min [68]. Concurrently, after a single running session in mice, an immediate increase in extracellular vesicle quantity was observed in both serum and brain tissue, which returned to pre-exercise levels within 90 min post-exercise [77]. In healthy males, following an exercise regimen consisting of eight sets of cycling at 140% VO_2max_ for 20 s with 10 s rest intervals, a significant elevation in the levels of EVs in serum was observed immediately post-exercise. However, there was a noticeable decline in EV quantity at 30 min and 120 min post-exercise [78]. EVs are considered to be an exercise factor, and the increase in their release is thought to reflect an adaptive response of the body to physical activity. Consequently, in order to maintain elevated levels of exosome release to facilitate the organism’s adaptation to exercise and to exert cardioprotective effects, sportspeople should pay attention to the duration of each exercise session and also maintain a long-term habit of physical activity. Furthermore, the regulation of cardiovascular diseases through exercise-mediated EVs is associated not only with the quantity of their release but also with the types of constituents they contain. The following text will elucidate the regulatory effects and mechanisms of EVs secreted during exercise of varying intensities on cardiovascular diseases.

## 5. Regulation of Cardiovascular Disease by EVs Secreted by Exercise of Different Intensities

### 5.1 Ischemic Stroke

Stroke is the second leading risk factor for disability and mortality in humans [79, 80]. Among these, ischemic stroke constitutes 87% of stroke incidence. Physical activity exerts both preventive and ameliorative effects on ischemic stroke by releasing EVs and altering their contents. Wang *et al*. [28] demonstrated that four weeks of moderate-intensity treadmill exercise before mice stroke modeling significantly reduced brain infarct volume, apoptotic cell rate, and caspase-3 clearance capability, while also increasing cerebral microvascular density. Further research indicated that exercise promoted the secretion of EVs derived from EPCs and the enrichment of miR-126 within these exosomes, which protected against ischemic injury in mice. Concurrently, additional research demonstrated that miR-126-3p within EVs mediated the recovery from ischemic stroke facilitated by a single session of high intensity interval cycling exercise [11, 81]. A 4-week treadmill exercise intervention in rats with ischemic stroke revealed a significant decrease in miR-338 levels in the serum following analysis of the contents of exosomes. The reduction in miR-338 expression regulated the hypoxia-inducible factor alpha (HIF-α) pathway, thereby protecting cerebral microvascular endothelial cells from the damage induced by ischemic stroke [82]. Eight weeks of moderate-intensity exercise increased the levels of miR-27a in the EVs secreted by EPCs, which alleviated oxidative stress by regulating mitochondrial membrane potential and then protecting cerebral neuroblastoma cells in hypertensive mice from damage caused by angiotensin II and hypoxic conditions [83]. Barcellos *et al*. [70] demonstrated that elderly rats subjected to 12 weeks of treadmill exercise had enhanced neurological recovery following stroke, as evidenced by elevated BDNF expression in plasma EVs. Study demonstrated that 8 weeks of high intensity interval training results in an enrichment of miR-223 in the EVs of obese individuals [75]. MiR-223 not only protected the brain from ischemia-induced neuronal death following a stroke by inhibiting calcium influx in hippocampal neurons through the suppression of glutamate receptor 2 (GluR2) and N-methyl-D-aspartate receptor 2B (NR2B), but also mitigated inflammation by targeting the NLRP3 inflammasome [80, 84]. The analysis of exosomes immediately extracted from the plasma of healthy subjects after a single bout of high intensity interval training revealed significant increases in the levels of miR-1-3p, miR-16-5p, miR-222-3p, miR-23a-3p, miR-208a-3p, and miR-150-5p [11]. Nevertheless, the elevated expression of miR-1-3p and miR-16-5p may represent a risk factor for the development of secondary cardiovascular diseases, while high levels of miR-222-3p have a detrimental impact on post-stroke recovery [10, 12]. The preceding analysis indicates that moderate and high intensity interval exercise primarily promote the recovery of ischemic stroke by regulating the expression of microRNA (miRNA) in EVs. However, high intensity continuous exercise may have adverse effects on the cerebrovascular systems. For example, Doncheva *et al*. [85] found that after a single 45-minute acute exercise session at 70% VO_2max_ in adult males, the levels of miR-222-3p in plasma EVs significantly increased. MiR-222-3p can induce the injury of human brain microvascular endothelial cells and increased expression of miR-222-3p is detrimental to recovery following a stroke [10].

### 5.2 Atherosclerosis

Atherosclerosis (AS) is the pathophysiological basis of cardiovascular diseases, and damage to the endothelium is an important early event leading to AS [35, 86, 87]. It is of paramount importance to maintain endothelial cell homeostasis as well as endothelial integrity in order to prevent the development of cardiovascular diseases such as AS. The study revealed that EVs derived from mouse skeletal muscle post-exercise are enriched with proteins involved in mitochondrial biogenesis and fatty acid β-oxidation. These EVs can be absorbed by metabolic tissue cells, such as adipocytes and hepatocytes, which regulate metabolic disorders and alleviate the occurrence and development of AS [35]. Low-intensity exercise has been demonstrated to stimulate the release of EVs containing miR-126 from EPCs [27]. MiR-126 can induce the recruitment of EPCs to injury sites through the stromal cell-derived factor-1 (SDF-1) in rats, thereby maintaining endothelial integrity [81]. Lou *et al*. [46] found that 9 days of moderate-intensity treadmill exercise promoted the aggregation of miR-122-5p in mouse hepatogenic EVs, and miR-122-5p could facilitate endothelial cell migration, thus aiding in the repair of damaged endothelium and other tissues. Moreover, patients with chronic coronary syndrome (CCS) exhibited a restored endothelialization capacity of EVs following 4 weeks of high intensity interval exercise, indicating that exercise may ameliorate CCS caused by AS through EVs [88]. Guescini *et al*. [4] demonstrated that exercise increased miR-181a-5p levels in plasma EVs of male students after a single 80% VO_2max_ high intensity running exercise intervention. It has been demonstrated that miR-181a-5p can mitigate endothelial activation and alleviate vascular inflammation through the inhibition of nuclear factor κB (NF-κB), thereby exerting anti-atherosclerotic effects [89]. The aforementioned studies indicate that EVs generated by exercise of varying intensities exert anti-atherosclerotic effects through mechanisms such as the inhibition of endothelial cell inflammation and the maintenance of endothelial integrity, with miRNAs playing a crucial mediating role therein.

### 5.3 MI/R Injury

MI/R can inflict serious damage to the structure and function of cardiomyocytes. Prevention of MI/R injury is particularly important for the prognosis of patients with myocardial infarction. Following a single moderate-intensity cycling intervention in both young and elderly individuals, there was an increase in nicotinamide phosphoribosyltransferase (Nampt) levels in their EVs, with a particularly significant elevation observed in the young population [71]. Nampt is a rate-limiting enzyme, the expression of which is significantly reduced when MI/R injury occurs, and up-regulation of Nampt enhances nicotinamide adenine dinucleotide (NAD^+^) and adenosine triphosphate (ATP) to inhibit reperfusion injury during myocardial ischemia [90]. The EVs generated by acute moderate-intensity endurance exercise induced nuclear translocation of nuclear factor erythroid 2-related factor 2 (Nrf2) and phosphorylation of heat shock protein 27 (HSP27) in cardiomyocytes, exerting antioxidant effects, thereby conferring protective effects on the heart [91]. Moreover, studies have demonstrated that high intensity exercise induces a substantial increase in brown adipose tissue in mice [92, 93, 94]. This process protects the heart from MI/R injury by secreting EVs enriched in microRNAs, including miR-125b-5p, miR-128-3p, and miR-30d-5p to inhibit the pro-apoptotic MAPK pathway [8]. Elevated levels of catalase (CAT) in serum EVs were found in young men following an acute high intensity interval exercise intervention [78], and specific overexpression of CAT in the heart has a cardioprotective effect on the heart by modulating autophagy, iron death, and oxidative stress in cardiomyocytes [95]. In conclusion, exercise of different intensities can play a role in cardioprotection and prevention of myocardial ischemia/reperfusion injury by regulating the expression of proteins and miRNAs in EVs.

## 6. Clinical Applications of Exercise-Induced EVs in Cardiovascular Protection

### 6.1 EVs-Containing miRNAs as Potential Biomarkers for Diagnosis of Cardiovascular Diseases 

MiRNAs are one of the most widely studied cargos of EVs in exercise regulating cardiovascular function. The expression of miRNAs can be modulated by cardiovascular diseases. Altered miRNA expression have been found in stroke, acute myocardial infarction, heart failure and other cardiovascular diseases [96]. In stroke patients, miR-145 and miR-21 levels were significantly higher compared with that in healthy controls [97]. MiR-208, miR-499 and miR-1 expressions were remarkably upregulated in acute myocardial infraction patients [98, 99, 100]. MiR-29b and miR-455-1 levels were positively correlated with the myocardial fibrosis and myocyte uncoupling [60]. The cardiovascular system is extremely sensitive to the changes in miRNAs levels [96]. Meanwhile, EVs are important sources and major transportation vehicles of miRNAs. Therefore, EVs containing miRNAs might become potential biomarkers for cardiovascular diseases. In addition, exercise exerts cardiovascular protection through increasing the expression of miR342-5p, miR126, miRNA-125b-5p, miR-445, miR-122-5p and other miRNAs [8, 26, 27, 46, 60]. These novel miRNAs might serve as biomarkers of exercise effectiveness in the exercise rehabilitation of cardiovascular diseases. In brief, EVs containing miRNAs are expected to be potential biomarkers for the predication, diagnosis, and therapy of cardiovascular diseases.

### 6.2 Exercise-Induced EVs as a Potential Strategy for the Therapies of Cardiovascular Diseases

Exercise improve cardiovascular function through altering the contents of EVs (Table 2, Ref. [8, 11, 25, 26, 28, 35, 46, 60, 70, 71, 78, 81, 82, 83, 91]), suggesting the potential therapeutic role of exercise-induced EVs in cardiovascular diseases. Emerging animal studies have confirmed the therapeutic effects of exercise-induced EVs [33, 81]. Liu *et al*. [33] isolated the circulating EVs from the plasma of rats subjected to 4 weeks of moderate aerobic exercise. The EVs were then added to human umbilical vein endothelial cells *in vitro* and cutaneous wounds in diabetic rats, respectively. The results demonstrated that EVs promoted angiogenesis and repair of skin defects, indicating that exercise-induced circulating EVs could be utilized as a therapy to active angiogenesis and diabetic wound healing. Alehossein *et al*. [81] found that treatment of sedentary mice with exosomes isolated from the plasma and the muscle of high intensity interval trained mice ameliorated glucose tolerance, insulin sensitivity, and reduced plasma levels of triglycerides. At present, the evaluation of therapeutic effects of exercise-induced EVs on cardiovascular protection has been focused on animal studies. Further human clinical trials are needed to confirm the effects of exercise-induced EVs for targeted therapy. Maybe, collecting EVs from healthy people or long-term exercisers and injecting into cardiovascular patients could be trialled, which may become a new approach for treating cardiovascular diseases. In addition, Liu *et al*. [101] uncovered that long-term caloric restriction alleviated aging-related-fibrosis of kidney through downregulation of miR-21 in EVs. Fasting regulated energy metabolism by promoting the secretion of EVs from adipose tissue [102]. Therefore, engineered EVs containing specific miRNAs or proteins could be constructed to mimic the effects of exercise or the other useful interventions, which may make cell-free targeted therapy for cardiovascular and other diseases possible.

**Table 2.** **The main research on the promotion of cardiovascular health by exercise-induced EVs**.

Research subjects	Exercise protocols			EVs cargo changes	Functional changes	Literature sources
	Intensity	Duration and frequency	Forms			
Healthy adults (n = 3)	50% VO_2max_	One time, 45 min	Treadmill running	SOD3↑	Promoting angiogenesis in endothelial cells	[25]
Healthy men (n = 21)	70% HR_max_	One time, 30 min	Treadmill running	MAP2K1↑	Protecting cardiomyocytes from oxidative stress	[91]
Healthy men (n = 10)	60 s of exercise, with a 75 s rest in between, repeated for a total of 10 sets	One time	Cycling	miR-126-3p↑	Protecting endothelial cells from hypoxic damage	[11]
Healthy men (n = 40)	70% VO_2max_	20 min	Cycling	Nampt↑	Suppressing myocardial cell apoptosis and promoting their survival	[71]
Young man (n = 17)	140% VO_2max_ for 20 s and rest for 10 s in between, repeated for a total of 8 sets	4 min	Cycling	CAT↑	Providing protective effects on the heart	[78]
8–10 week-old C57BL/6J mice	5 m/min	60 min/d, 5 d/w, 4 w	Treadmill running	miR-126↑	Inducing the recruitment of EPCs to the site of injury to maintain endothelial integrity	[81]
*db/db* mice	7 m/min	300 m/d, 8 w	Treadmill running	miR-445↑, miR-29b↑	Preventing myocardial fibrosis and uncoupling	[60]
Adult male	6.59 m/min	40 min/d, 6 d/w, 4 w	Wheel training	miR-338↓	Protecting brain microvascular endothelial cells	[82]
Sprague-Dawley (SD) rats performed transient middle cerebral artery occlusion surgery						
8 week-old C57BL/6J mice	18 m/min	60 min/d, 9d	Treadmill running	miR-122-5p↑	Enhancing endothelial cell fatty acid utilization to promote angiogenesis	[46]
7–8 week-old hypertensive transgenic mice	10 m/min	60 min/d, 5 d/w,8 w	Treadmill running	miR-27a↑	Suppressing ROS production in N2a cells to prevent damage caused by oxidative stress	[83]
8–10 week-old C57BL/6J mice	10 m/min	5 d/w, 4 w	Treadmill running	miR-126↑	Decreasing cell apoptosis	[28]
2-month-old and 22-month-old Wistar rats	60% VO_2max_	20 min/time, 3 times/w, 12 w	Aerobic, acrobatic, resistance and combined	BDNF↑	Promoting the recovery of neurological function after stroke	[70]
8–10 week-old C57BL/6J mice	-	30 min/d, 4 w	Swimming	-	Alleviating atherosclerosis in *ApoE*-deficient mice	[35]
6–8 week-old C57BL/6J mice	-	90 min/d, 2 times/d, 4 w	Swimming	miR-125-5p↑ miR-128-3p↑ miR-30d-5p↑	Protecting the heart from myocardial ischemia/reperfusion injury	[8]
6 week-old SD rats	-	90 min/d, 7 times/w, 4 w	Swimming	miR342-5p↑	Protecting the heart against myocardial ischemia/reperfusion	[26]

Notes: SOD3, extracellular superoxide dismutase; MAP2K1, mitogen-activated protein kinase kinase 1; Nampt, nicotinamide phosphoribosyltransferase; CAT, catalase; BDNF, brain-derived neurotrophic factor; EPCs, endothelial progenitor cells; ROS, reactive oxygen species; N2a cell, mouse neuroblastoma N2a cells; ApoE, apolipoprotein E; EVs, extracellular vesicles; VO_2max_, maximal oxygen uptake; HR_max_, maximal heart rate; d, day; w, week; m, minute. ↑ indicates increase, ↓ indicates decrease.

## 7. Discussions and Conclusions

**Fig. 1.**
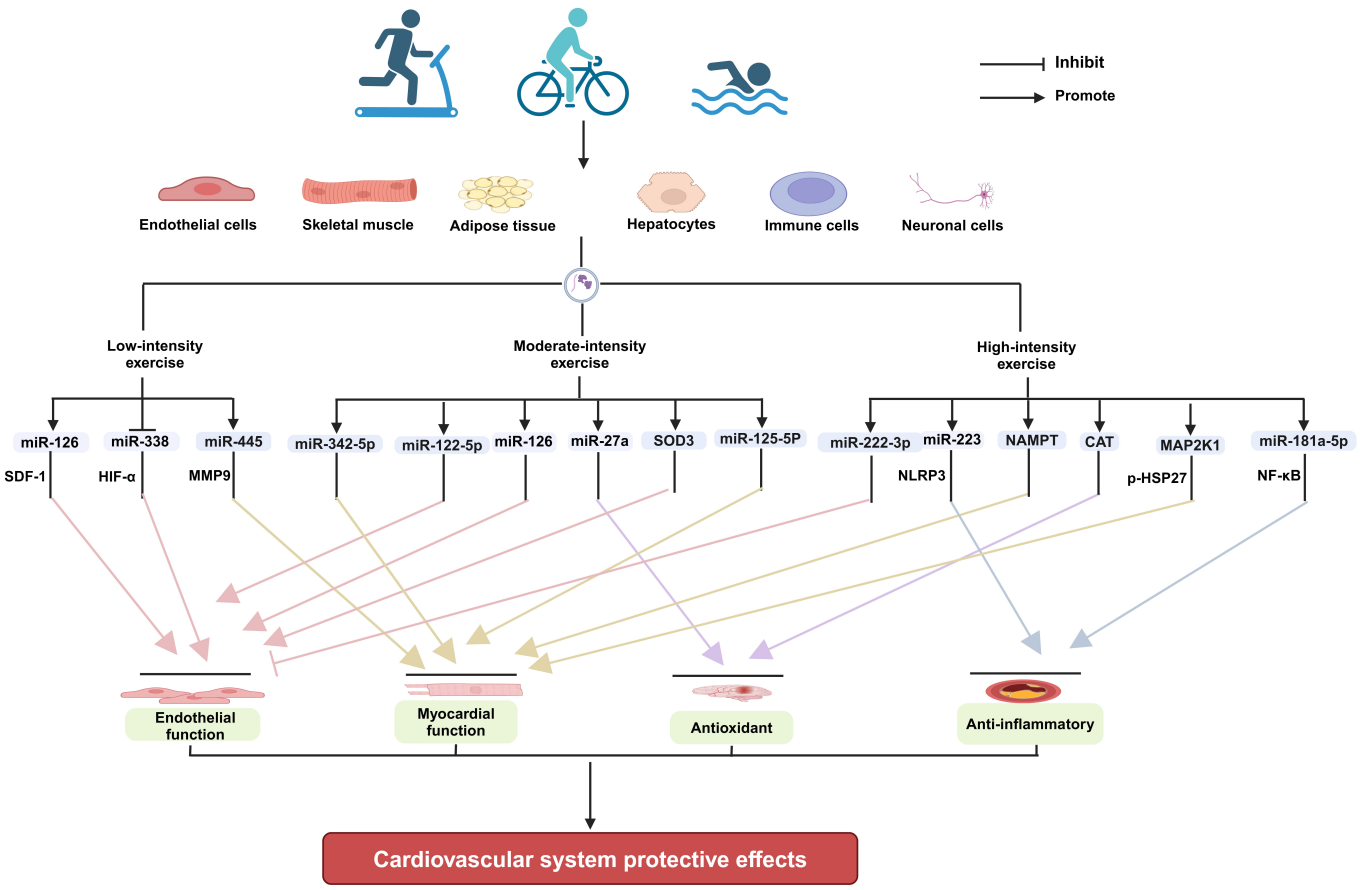
**The mechanisms underlying the cardioprotective effects of EVs produced in response to exercise of varying intensities (Figure created with BioRender.com)**. EVs, extracellular vesicles; SOD3, extracellular superoxide dismutase; CAT, catalase; MAP2K1, mitogen-activated protein kinase kinase 1; SDF-1, stromal cell-derived factor-1; HIF-α, hypoxia-inducible factor alpha; MMP9, matrix metallopeptidase 9; NLRP3, NOD-like receptor family pyrin domain-containing 3; HSP27, heat shock protein 27; NF-κB, nuclear factor κB; Nampt, nicotinamide phosphoribosyltransferase.

Exercise improving cardiovascular health is a systemic and integrative effect. Exercise can produce direct effects locally in tissues and organs, such as enhancing muscle strength, increasing stroke volume, promoting angiogenesis, and raising insulin sensitivity [103, 104]. Meanwhile, exercise can also elicit the secretion of cardioprotective exerkines, such as miRNAs from multiple tissues and organs of body, mediating tissue-organ interactions by endocrine and paracrine means [26, 105, 106]. The direct and indirect effects collectively contribute to cardiovascular health. EVs are important transportation vehicles for miRNAs and other types of exerkines. Exercise of different intensities can modulate the release of EVs with biological functions from specific tissue cells, such as endothelial cells, skeletal muscle, and adipose tissue, and exert a protective effect on the cardiovascular system by altering their contents (Fig. 1).

Current research generally suggests that all intensities of exercise are capable of increasing the secretion of EVs in serum/plasma in different populations, except for high intensity exercise performed by populations with cardiovascular disease or cardiovascular risk [32, 68, 71]. Moreover, in addition to high intensity continuous exercise, low, moderate and high intensity interval exercise can improve cardiovascular function through exercise-induced EVs [25, 60, 91, 107]. However, there is limited research on the regulatory law of EV secretion in response to low, moderate, and high intensities exercise, and comprehensive studies are needed. Meanwhile, at present, most studies are looking at plasma/serum samples, where it is very difficult to parse out differences between EVs and the proteins in the plasma/serum. It is well known that there are exercise-related proteins changes in blood [108]. These plasma proteins form protein corona of EVs, which may or may not be removed from the plasma depending on current EVs isolation methods, such as size-exclusion chromatography (SEC) and the ultracentrifuge method [109]. Therefore, the role of protein corona is ignored. It is also difficult to determine whether inner cargos of EVs (proteins, nucleic acids, lipids, etc.) or protein corona are playing a major role in improving cardiovascular diseases. In addition, for the inner cargo of EVs, each study identifies their changes, but these are generally poorly reproduced between studies, especially the studies with same exercise intensity. We may understand that exercise-secreted EVs exerting a cardioprotective function are controlled by multiple signaling pathways. All these proteins or nucleic acids play effective roles. However, their roles maybe not equal. Which protein or nucleic acid plays a key role remains to be determined. This is particularly important for precise treatment in cardiovascular diseases by simulating the effects of exercise in clinic. Therefore, there is still a long way to go through using exercise-secreted EVs or constructing engineered EVs mimicking the effects of exercise for clinical applications in cardiovascular or other diseases.

## Abbreviations

EVs, extracellular vesicles; ESEs, early-sorting endosomes; LSEs, late-sorting endosomes; MVBs, multivesicular bodies; Akt, protein kinase B; Jnk2, c-Jun N-terminal kinase 2; EPCs, endothelial progenitor cells; HSP60, heat shock protein 60; FNDC5, fibronectin type III domain-containing protein 5; MAPK, mitogen-activated protein kinase; MI/R, myocardial ischemia-reperfusion; LAMP1, lysosomal associated membrane protein 1; AGPAT1, 1-acyl-sn-glycerol-3-phosphate acyltransferase; VO_2max_, maximal oxygen uptake; HR_max_, maximal heart rate; HRR, heart rate reserve; 1 RM, percentage of one repetition maximum; MMP9, matrix metallopeptidase 9; ELVs, exosome-like vesicles; HIF-α, hypoxia-inducible factor alpha; BDNF, brain-derived neurotrophic factor; GluR2, glutamate receptor 2; NR2B, N-methyl-D-aspartate receptor 2B; NLRP3, NOD-like receptor family pyrin domain-containing 3; AS, atherosclerosis; SDF-1, stromal cell-derived factor-1; CCS, chronic coronary syndrome; NF-κB, nuclear factor κB; Nampt, nicotinamide phosphoribosyltransferase; NAD^+^, nicotinamide adenine dinucleotide; Nrf2, nuclear factor erythroid 2-related factor 2; HSP27, heat shock protein 27; miRNAs, microRNAs; CAT, catalase; VEGF, vascular endothelial growth factor; JAK, Janus tyrosine kinase; STAT, signal transducer and activator of transcription; AD, Alzheimer’s disease; CASP2, caspase 2; *ApoE*^-/-^, apolipoprotein E knock out; SOD3, extracellular superoxide dismutase; SD rats, Sprague-Dawley rats; ROS, reactive oxygen species; N2a cell, mouse neuroblastoma N2a cells; mPTP, mitochondrial permeability transition pores; MAP2K1, mitogen-activated protein kinase kinase 1; SEC, size-exclusion chromatograph; *TSG101*, tumor susceptibility gene; ATP, adenosine triphosphate.
